# Risankizumab Induction Therapy Achieves Early Symptom Improvements That Are Associated With Future Clinical and Endoscopic Outcomes in Crohn’s Disease: Post Hoc Analysis of the ADVANCE, MOTIVATE, and FORTIFY Phase 3 Studies

**DOI:** 10.1093/ecco-jcc/jjad206

**Published:** 2023-12-09

**Authors:** Jean-Frederic Colombel, Stefan Schreiber, Geert D’Haens, Joanne Rizzo, Kristina Kligys, Jenny Griffith, Javier Zambrano, Qian Zhou, Yafei Zhang, Jasmina Kalabic, Florian Rieder, Marla C Dubinsky, Remo Panaccione

**Affiliations:** The Henry D. Janowitz Division of Gastroenterology, Department of Medicine, Icahn School of Medicine at Mount Sinai, New York, NY, USA; Department of Medicine I, University Hospital Schleswig-Holstein, Christian-Albrechts-University, Kiel, Germany; Department of Gastroenterology, Amsterdam University Medical Center, Amsterdam, The Netherlands; AbbVie Inc, North Chicago, Illinois, USA; AbbVie Inc, North Chicago, Illinois, USA; AbbVie Inc, North Chicago, Illinois, USA; AbbVie Inc, North Chicago, Illinois, USA; AbbVie Inc, North Chicago, Illinois, USA; AbbVie Inc, North Chicago, Illinois, USA; AbbVie Deutschland GmbH & Co KG, Ludwigshafen, Germany; Department of Gastroenterology, Hepatology & Nutrition, Inflammation and Immunity, Cleveland Clinic, Cleveland, Ohio, USA; Susan and Leonard Feinstein IBD Center, Icahn School of Medicine at Mount Sinai, New York, NY, USA; Inflammatory Bowel Disease Unit, Division of Gastroenterology and Hepatology, University of Calgary, Calgary, Alberta, Canada

**Keywords:** Risankizumab, Crohn’s disease, patient-reported outcomes, early symptom improvement

## Abstract

**Background and Aims:**

Crohn’s disease [CD] symptoms are a main driver for impaired quality of life, and fast relief is important for patient care. Stool frequency [SF] and abdominal pain score [APS] are patient-reported outcomes [PROs] measuring symptom severity, which are supported as treatment targets by the STRIDE-II consensus. This post hoc analysis examined the efficacy of risankizumab [RZB], a humanised monoclonal antibody with high specificity for interleukin-23 p19, for providing early symptom relief, along with the prognostic value of early symptom relief for achieving future clinical and endoscopic endpoints.

**Methods:**

Individual and combined measures of SF and AP at Weeks 1, 2, and 3 were assessed in patients with moderate to severe CD who received 600 mg intravenous RZB or placebo [PBO] in the ADVANCE or MOTIVATE induction studies. Multivariate logistic regression was used to examine the predictiveness of early symptom improvement for clinical and endoscopic outcomes following RZB induction and maintenance.

**Results:**

Higher rates of SF/APS clinical remission and enhanced clinical response were observed as early as Week 1 with RZB vs PBO. A larger proportion of patients achieved clinical endpoints with RZB vs PBO, irrespective of prior bio-failure status. Early PRO improvement was associated with a greater likelihood of achieving clinical and endoscopic improvement following 12-week induction and 52-week maintenance RZB dosing.

**Conclusions:**

After the first intravenous RZB induction dose, significantly greater rates of symptom improvement vs PBO were achieved. Improvements could be observed as early as Week 1 and were predictive of Weeks 12 and 52 clinical and endoscopic improvement.

## 1. Introduction

Crohn’s disease [CD] is a chronic, progressive, inflammatory disease affecting the gastrointestinal tract, characterised by the hallmark symptoms of chronic diarrhoea, abdominal pain, and fatigue.^1^ These symptoms greatly affect daily life, including work, education, and social relationships, and are associated with increased anxiety, depression, and impaired health-related quality of life [hrQoL].^2,3^ For patients with CD, relief of symptoms is an important proximate treatment goal.^4^

Patient-reported outcome [PRO] measures for the two most prominent and burdensome symptoms of CD, as measured by the weighted daily stool frequency [SF] and abdominal pain [AP] items from the Crohn’s Disease Activity Index [CDAI], are increasingly being included in clinical trials to evaluate symptomatic relief.^5–7^ Current [STRIDE-II] recommendations suggest targeting clinical response (defined as at least a 50% reduction in SF and AP scores [APS]) as an immediate treatment target, and clinical remission [SF ≤2.8–3.0 and APS ≤1] as an intermediate-term treatment target, for which intensification or modification of treatment should be considered if not achieved.^8^ Importantly, clinical remission, [SF ≤2.8 and APS ≤1.0], and clinical response [≥30% decrease in SF or APS] have been correlated with higher scores of general wellbeing and greater improvements in wellbeing, respectively, in patients with moderate to severe CD.^9^

Risankizumab [RZB] is a humanised immunoglobulin G1 monoclonal antibody that inhibits interleukin-23 by binding with high specificity to its p19 subunit.^10^ RZB induction therapy met the co-primary outcomes of clinical remission and endoscopic response at week 12 in patients with moderately to severely active CD, in the double-blind, randomised, placebo [PBO]-controlled phase 3 studies ADVANCE and MOTIVATE, with clinical response/remission observed as early as week 4.^11^ This post hoc analysis examined early symptom relief at weeks 1 through 3, as measured by the PROs of SF and APS following the first dose of intravenous [IV] RZB induction vs PBO during ADVANCE and MOTIVATE. In addition, the association between achievement of early symptom improvement and the likelihood of achieving later clinical and endoscopic endpoints was analysed.

## 2. Methods

### 2.1. Study design and treatment

The ADVANCE [NCT03105128] and MOTIVATE [NCT03104413] studies were two parallel phase 3, double-blind, randomised, PBO-controlled, 12-week induction trials in patients with moderately to severely active CD. FORTIFY [NCT03105102] Sub-study 1 [S-S1-] was a 52-week phase 3, double-blind, randomised, responder withdrawal study evaluating efficacy and safety of continuing RZB as SC maintenance therapy vs withdrawal of RZB therapy [PBO], in patients with clinical response to 12 weeks of intravenous [IV] RZB induction treatment. Detailed descriptions of ADVANCE, MOTIVATE, and FORTIFY study designs, participants, randomisation, procedures, outcome measures, and statistical analyses were previously reported in the primary induction and maintenance manuscripts.^11,12^ Here, methods relevant to this post hoc analysis are described. Briefly, patients were randomised 2:1 [ADVANCE] or 1:1 [MOTIVATE] to receive IV RZB 600 mg or PBO IV at baseline, week 4, and week 8. Clinical responders to RZB induction entering FORTIFY SS1 were re-randomised 1:1:1 to receive RZB 180 mg subcutaneously [SC], RZB 360 mg SC, or PBO (withdrawal [PBO SC]) every 8 weeks.

### 2.2. Patients

ADVANCE and MOTIVATE were randomised, double-blinded, PBO-controlled, phase 3 induction studies. Eligible patients were aged 16–80 years, with moderately to severely active CD. All patients in MOTIVATE had a history of bio-failure, defined as an intolerance or inadequate response to one or more approved biologics for CD. Patients in ADVANCE may have had prior bio-failure and/or an intolerance or inadequate response to conventional therapy (aminosalicylates, oral, locally acting steroids [eg, budesonide, beclomethasone], systemic corticosteroids [prednisone or equivalent], or immunomodulators).

### 2.3. Assessments

#### 2.3.1. Efficacy

PROs were collected from an electronic diary, a handheld device into which the patient recorded required information on a daily basis. During the screening visit, patients were trained on how to complete the electronic diary by site staff, and the diary was reviewed by site personnel at each visit. Average daily SF and average daily APS were calculated based on the number of very soft and liquid stools and on abdominal pain rating [0 = none, 1 = mild, 2 = moderate, 3 = severe], respectively. Data from seven of the most recent useable days out of the 14 days preceding the visit were used to calculate PROs. If seven useable days were not available, an average was calculated as follows. If there were non-missing diary data from less than 7 days but greater than 3 days [4, 5, 6 days] OR from 3 consecutive days, the subtotal score was calculated as average of the diary data × 7 × factor [2 for SF and 5 for AP]. If a minimum number of 4 days, or 3 consecutive days, of diary data were not available, the patient’s score for that visit was considered missing [Supplementary Table 1]. The CDAI (Crohn’s Disease Activity Index) was calculated in order to evaluate deep remission (CDAI <150) and endoscopic remission (Simple Endoscopic Score for Crohn’s Disease [SES-CD] ≤4), at least a 2-point reduction vs baseline of the induction study, and no subscore greater than 1 in any individual variable, as scored by a central reviewer]).

Symptom improvement over the first 3 weeks of induction treatment was evaluated via the following: achievement of SF remission [defined as SF ≤2.8]; AP remission [defined as APS ≤1]; absolute change [decrease] from baseline in SF; absolute change [decrease] from baseline in APS; SF/APS clinical remission [defined as SF ≤2.8 and APS ≤1]; and enhanced clinical response (defined as ≥60% decrease in average daily SF and/or ≥35% decrease in average daily APS [and both not worse than baseline]; and/or SF/APS clinical remission). Outcomes were also examined according to bio-failure status [with or without prior bio-failure] as well as disease location [colonic or ileal].

#### 2.3.2. Safety

The safety analysis population included all patients who received ≥1 dose of study drug [PBO, RZB 600 mg IV, RZB 180 mg SC, RZB 360 mg SC]. Safety assessments included measuring tolerability and incidence of adverse events.

### 2.4. Statistical analysis

Efficacy analyses were conducted in the intention-to-treat [ITT] population, defined as randomised patients who had received at least one dose of study drug [RZB 600 mg IV, PBO] during the 12-week induction period; the primary population for efficacy analysis were patients in the ITT analysis set who had baseline eligibility SES-CD of ≥6 [≥4 for isolated ileal disease], excluding patients from a noncompliant site [*n* = 3, ADVANCE; *n *= 9, MOTIVATE]. Results for categorical endpoints are based on non-responder imputation for missing data. Treatment differences are based on the Cochran–Mantel–Haenszel test for categorical endpoints and the mixed-effect model repeated measurement model for continuous endpoints.

Pooled data from patients who received RZB 600 mg intravenously [IV] in the ADVANCE + MOTIVATE induction studies (*n *= 527; with prior bio-failure: *n* = 386 [73.2%]; without prior bio-failure: *n* = 141 [26.8%]) were evaluated to determine the predictiveness of early achievement of PRO-based endpoints for achieving clinical and endoscopic outcomes, following induction and maintenance dosing. Separate logistic regression models were used with each early symptom outcome as a fixed effect in the model. Odds ratios, along with associated 95% confidence intervals [CIs] [two-sided] from the model were provided. For FORTIFY, separate logistic regression models were used to access end-of-induction characteristics for the achievement of outcomes at week 52.

## 3. Results

### 3.1. Efficacy

#### 3.1.1. Early symptom changes from baseline—total population

**Figure 1:**
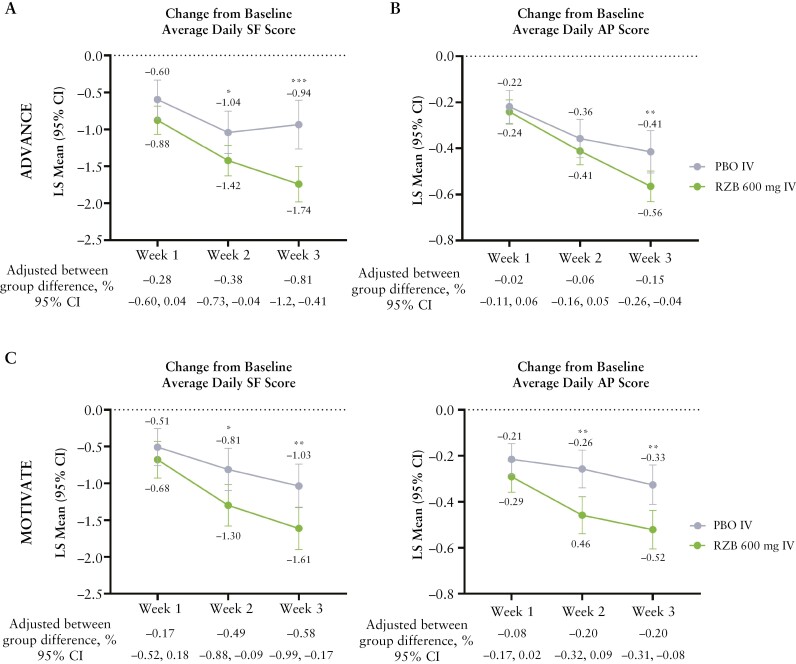
Change from baseline in average daily SF or AP score over time [ITT population, MMRM]. Week 1: ADVANCE—PBO, *N* = 168; RZB 600 mg, *N* = 320; MOTIVATE: PBO, *N* = 183; RZB 600 mg, *N *= 188; Week 2: ADVANCE—PBO, *N* = 164; RZB 600 mg, *N *= 324; MOTIVATE: PBO, *N *= 170; RZB 600 mg, *N* = 188; Week 3: ADVANCE—PBO, *N* = 159; RZB 600 mg, *N* = 319; MOTIVATE—PBO, *N* = 175; RZB 600 mg, *N* = 186; *p ≤0.05, **p ≤0.01, ***p ≤0.001 vs PBO. AP, abdominal pain; ITT, intention-to-treat; MMRM, mixed-effect model repeat measurement; PBO, placebo; RZB, risankizumab; SF, stool frequency. MMRM with the categorical fixed effects of treatment, visit and treatment-by-visit interaction, stratification factors (number of prior biologics failed [0, 1, >1] and baseline steroid use [Yes, No]), and the continuous fixed covariates of baseline measurements included in the model.

RZB treatment led to a significantly greater reduction in average daily SF [95% CI] from baseline as early as week 2 vs PBO in both ADVANCE [-1.4 vs -1.0; adjusted difference -0.38%; *p* ≤0.05; Figure 1A] and MOTIVATE [-1.3 vs -0.8; adjusted difference -0.49%; *p* ≤0.05; Figure 1C]. Similarly, a reduction of average daily APS with RZB vs PBO was observed as early as week 2, with significant changes observed in both ADVANCE [-0.57 vs -0.42; adjusted difference -0.15%; *p* ≤0.01; Figure 1B] and MOTIVATE [-0.5 vs -0.3; adjusted difference -0.20%; *p* ≤0.01; Figure 1D] by week 3.

**Figure 2:**
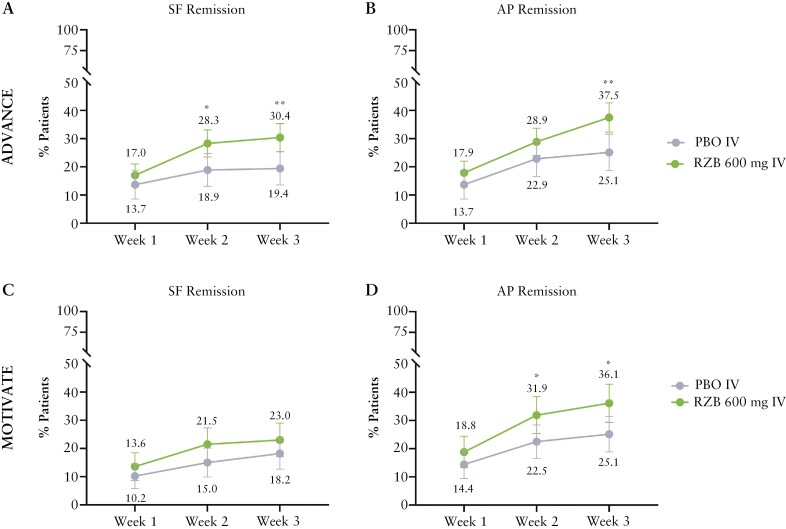
Achievement of SF or AP remission over time [ITT population, NRI-NC]. ADVANCE: PBO, *N* = 175; RZB 600 mg, *N *= 336; MOTIVATE: PBO, *N* = 187; RZB 600 mg, *N* = 191; *p ≤0.05, **p ≤0.01 vs PBO. AP remission,  average daily APS ≤1 and not worse than baseline; ITT, intention-to-treat population; NRI-NC, non-responder imputation with no special data handling for missing data, due to COVID-19; PBO, placebo; RZB, risankizumab; SF, stool frequency; SF remission, average daily SF ≤2.8 and not worse than baseline.

In ADVANCE and MOTIVATE, approximately 90% of patients had SF >2.8 and APS >1 at BL [Supplementary Table 2], compared with PBO. Patients who received 600 mg RZB IV in ADVANCE and MOTIVATE experienced numerically higher individual rates of SF remission and AP remission as early as week 1 post first RZB dose [Figure 2]. At week 2, a significantly greater proportion of patients in ADVANCE achieved SF remission with RZB [28.3%] vs PBO [18.9%; adjusted difference, 8.9%; *p* ≤0.05; Figure 2A], with even greater efficacy over PBO observed at week 3 [30.4% vs 19.4%; adjusted difference 10.9%; *p* ≤0.01]. Significantly higher rates of AP remission were also achieved with RZB vs PBO by week 2; by week 3, RZB-treated patients in both ADVANCE and MOTIVATE demonstrated significantly greater AP remission rates vs PBO [37.5% vs 25.1%; adjusted difference 11.6%; *p ≤*0.01 and 36.1% vs 25.1%; adjusted difference 11.1%; *p* ≤0.05, respectively] [Figure 2B and D]. The proportion of patients who had SF remission or AP remission is shown in Supplementary Table 2.

**Figure 3:**
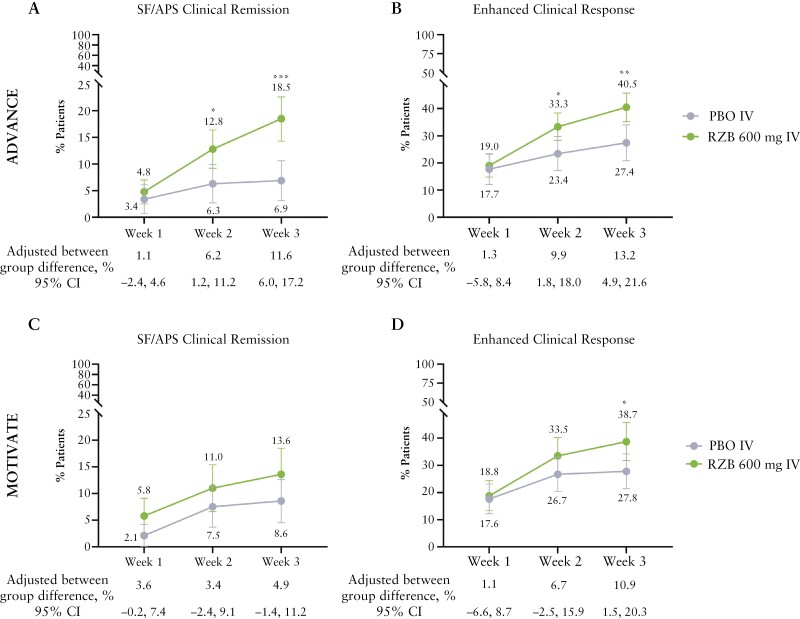
Achievement of SF/APS clinical remission, enhanced clinical response over time [ITT population, NRI-NC]. ADVANCE—PBO, *N* = 175; RZB 600 mg, *N* = 336; MOTIVATE:—PBO, *N* = 187; RZB 600 mg, *N* = 191; *p ≤0.05, **p ≤0.01, ***p ≤0.001 vs PBO. APS, abdominal pain score; enhanced clinical response per SF/APS criteria,  ≥60% decrease in average daily SF and/or ≥35% decrease in average daily AP [and both not worse than baseline] and/or clinical remission; ITT, intention-to-treat; NRI-NC, non-responder imputation with no special data handling for missing data, due to COVID-19; PBO, placebo; RZB, risankizumab; SF, stool frequency; SF/APS clinical remission, average daily SF ≤2.8 and average daily AP score ≤1 and both not worse than baseline.

Additionally, RZB treatment also led to numerically higher rates of the combined PRO endpoints of SF/APS clinical remission and enhanced clinical response at weeks 1 through 3, compared with PBO [Figure 3]. Significantly higher rates of SF/APS clinical remission [12.8% vs 6.3%; adjusted difference 6.2%, *p* ≤0.05; Figure 3A] and enhanced clinical response [33.3% vs 23.4%; adjusted difference 9.9%; *p* ≤0.05; Figure 3B] with RZB vs PBO were observed by week 2 in ADVANCE. At week 3, the proportion of patients achieving enhanced clinical response with RZB vs PBO was significantly greater in both ADVANCE [40.5% vs 27.4%; adjusted difference 13.2%; *p* ≤0.01; Figure 3B] and MOTIVATE [38.7% vs 27.8%; adjusted difference 10.9%; *p* ≤0.05; Figure 3D].

#### 3.1.2. Early symptom changes from baseline—with and without prior bio-failure

Subgroup analyses of patients with or without prior bio-failure in the ADVANCE study demonstrated numerically greater rates of AP remission and SF remission in the RZB group as early as week 1 post first induction dose of RZB vs PBO, irrespective of bio-failure status; numerically higher rates of efficacy, however, were observed for patients without prior bio-failure relative to patients with prior bio-failure [Supplementary Figure 1]. Rates of SF remission at week 2 were significantly greater with RZB vs PBO in patients without prior bio-failure [Supplementary Figure 1A] and, at week 3, rates of AP remission were significantly greater with RZB vs PBO in patients with prior bio-failure [Supplementary Figure 1D].

Subgroup analysis for the combined endpoint of SF/APS clinical remission demonstrated numerically greater rates with RZB treatment compared with PBO as early as week 2, irrespective of prior bio-failure status, with significantly greater rates observed with RZB at week 3 for both subgroups vs PBO [with prior bio-failure, 16.4% vs 8.2%; adjusted difference 8.0%; *p* ≤0.05; Supplementary Figure 2A; without prior bio-failure, 21.3% vs 5.1%; adjusted difference 16.1%; *p* ≤0.001; Supplementary Figure 2B]. For enhanced clinical response, 34.9% of patients with prior bio-failure, treated with RZB, achieved SF/APS enhanced clinical response at week 2 vs PBO [23.7%; adjusted difference 11.4%; *p* ≤0.05; Supplementary Figure 2C]; significant differences between RZB and PBO in patients without prior bio-failure were observed at week 3 [44.7% vs 26.9%; adjusted difference 17.7%; *p* ≤0.01; Supplementary Figure 2D].

#### 3.1.3. Early clinical outcomes as predictors of response

**Figure 4:**
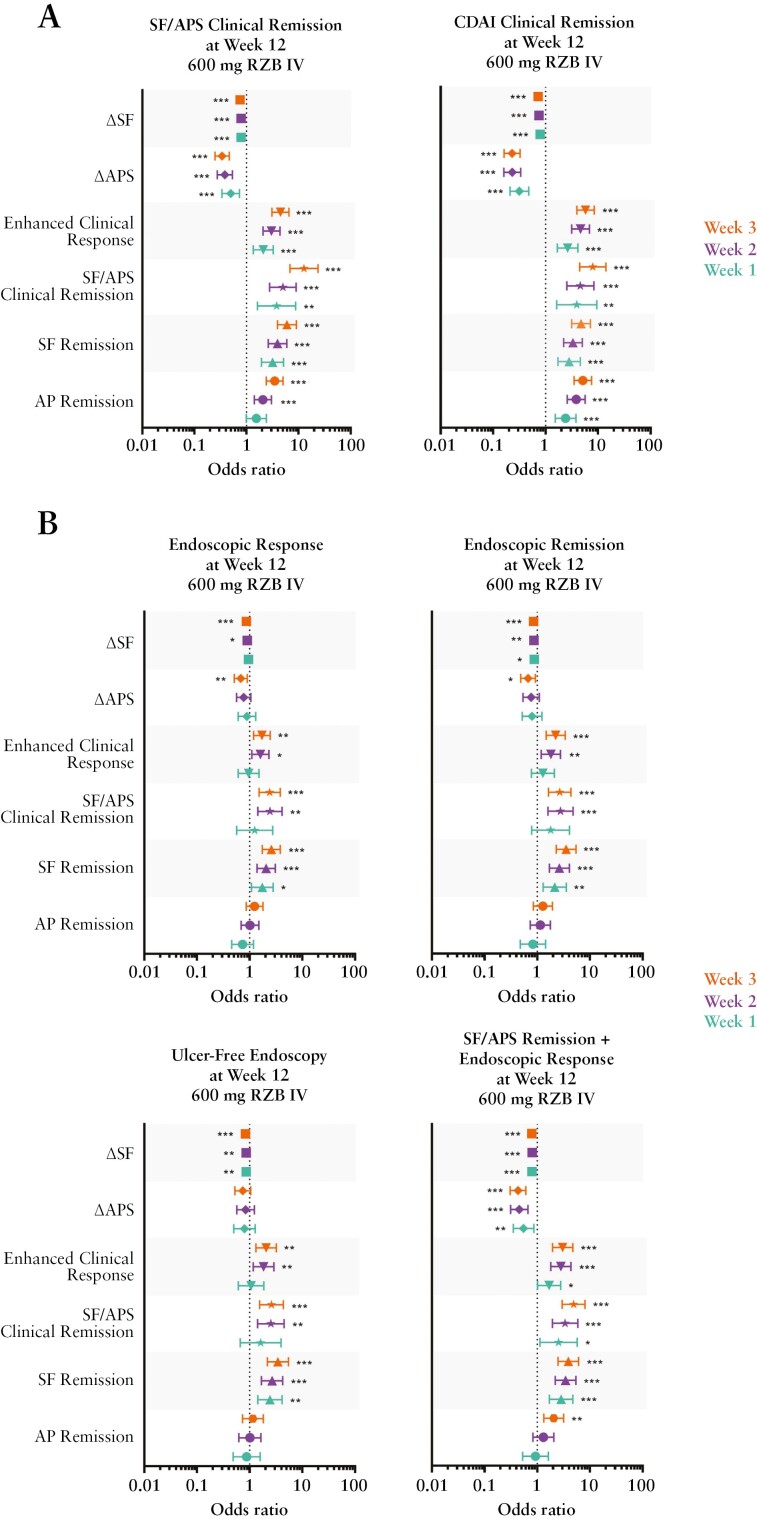
Early clinical outcomes as predictors of achieving endoscopic and/or symptomatic endpoints following induction. Pooled data from patients [*N* = 527] who received RZB 600 mg intravenous [IV] in the ADVANCE + MOTIVATE induction studies; a significant odds ratio less than 1 for ΔSF and ΔAPS is reflective of the fact that as the variable decreases, the event is more likely to occur. AP remission = average daily AP score ≤1 and not worse than baseline; SF remission, average daily SF ≤2.8 and not worse than baseline; SF/APS clinical remission, average daily SF ≤2.8 and not worse than baseline and average daily AP score ≤1 and not worse than baseline; CDAI clinical remission, CDAI <150; enhanced clinical response,  ≥60% decrease in average daily SF and/or ≥35% decrease in average daily AP score and both not worse than baseline, and/or clinical remission; endoscopic response, decrease in SES-CD >50% from baseline [or for subjects with isolated ileal disease and a baseline SES-CD of 4, at least a 2-point reduction from baseline], as scored by central reviewer; endoscopic remission, SES-CD ≤4 and at least a 2-point reduction vs baseline and no subscore greater than 1 in any individual variable, as scored by a central reviewer; ulcer-free endoscopy,  SES-CD ulcerated surface subscore of 0 in patients with SES-CD ulcerated surface subscore ≥1 at baseline, as scored by a central reviewer; *p ≤0.05, **p ≤0.01, ***p ≤0.001. SF, stool frequency; AP, abdominal pain; RZB, risankizumab; CDAI, Crohn’s Disease Activity Index; SES-CD, Simple Endoscopic Score for Crohn’s Disease.

In patients who achieved early symptom improvement, the likelihood of achieving clinical and endoscopic endpoints at week 12 of induction was analysed. Using pooled data from patients in ADVANCE + MOTIVATE [RZB 600 mg IV, *n *= 527], patients achieving early SF remission, a change from BL in SF, SF/APS clinical remission, or enhanced clinical response [as early as week 1] were found to be significantly more likely to achieve all symptomatic, endoscopic, and composite endoscopic/symptomatic outcomes examined at week 12 [Figure 4]. Achievement of AP remission or a decrease in APS from BL, as early as week 2, was associated with a significantly greater likelihood of achieving clinical remission [SF/APS and CDAI] at week 12 [Figure 4A]. A decrease in APS from BL was also associated with a significantly greater likelihood of achieving endoscopic response, endoscopic remission, and the composite endpoint of SF/APS clinical remission + endoscopic response at week 12 [Figure 4B].

**Figure 5:**
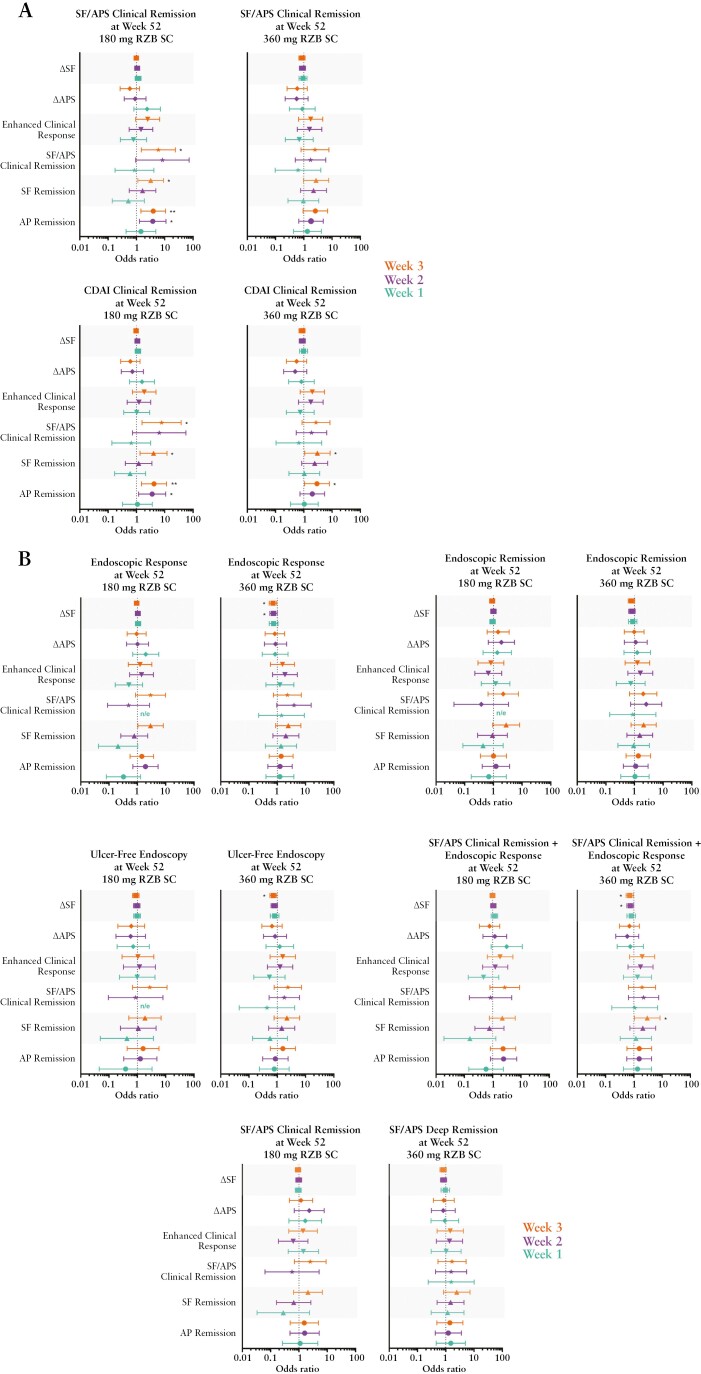
Early clinical outcomes as predictors of achieving clinical, endoscopic, and composite endpoints following maintenance dosing. RZB 180 mg SC, *N *= 69; RZB 360 mg SC, *N* = 65. A significant odds ratio less than 1 for ΔSF and ΔAPS is reflective of the fact that as the variable decreases, the event is more likely to occur. AP remission, average daily AP score ≤1 and not worse than baseline; SF remission, average daily SF ≤2.8 and not worse than baseline; SF/APS clinical remission, average daily SF ≤2.8 and not worse than baseline and average daily AP score ≤1 and not worse than baseline; CDAI clinical remission, CDAI <150; enhanced clinical response,  ≥60% decrease in average daily SF and/or ≥35% decrease in average daily AP score and both not worse than baseline, and/or clinical remission; endoscopic response, decrease in SES-CD >50% from baseline [or for patients with isolated ileal disease and a baseline SES-CD of 4, at least a 2-point reduction from baseline], as scored by central reviewer; endoscopic remission, SES-CD ≤4 and at least a 2-point reduction vs baseline and no subscore greater than 1 in any individual variable, as scored by a central reviewer; ulcer-free endoscopy, SES-CD ulcerated surface subscore of 0 in patients with SES-CD ulcerated surface subscore ≥1 at baseline, as scored by a central reviewer; SF/APS deep remission, SF/APS clinical remission + endoscopic remission; *p ≤0.05, **p ≤0.01. SC, subcutaneous; SF, stool frequency; APS, abdominal pain score; RZB, risankizumab; CDAI, Crohn’s Disease Activity Index; SES-CD, Simple Endoscopic Score for Crohn’s Disease.

In patients who received RZB 600 mg IV during induction and received either RZB 180 mg SC or RZB 360 mg SC maintenance in FORTIFY [RZB 180 mg SC, *n* = 69; RZB 360 mg SC, *n* = 65], achievement of AP remission and SF remission [as early as week 2] was associated with a significantly higher likelihood of achieving CDAI clinical remission at week 52 for both maintenance doses, and SF/APS clinical remission for the RZB 180-mg SC dose [Figure 5A]. Achievement of SF/APS clinical remission at week 3 was also associated with a significantly higher likelihood of achieving clinical remission [SF/APS or CDAI] at week 52 [Figure 5A] in patients receiving RZB 180 mg SC. With respect to endoscopic outcomes, early reduction in SF [weeks 2/3] was associated with a significantly greater likelihood of achieving endoscopic response, ulcer-free endoscopy, and the composite endpoint of SF/APS clinical remission plus endoscopic response at week 52, in patients receiving the 360 mg RZB SC dose. Achieving SF remission at week 3 was associated with a significantly greater likelihood of achieving endoscopic response at week 52 in patients receiving the 180 mg RZB SC dose [Figure 5B].

When examining patients with or without a history of prior bio-failure, improvement of PROs at weeks 1–3 were associated with a significantly increased likelihood of achieving clinical remission [SF/APS or CDAI] [Supplementary Figure 3A] and the combined endpoint of SF/APS clinical remission plus endoscopic response [Supplementary Figure 3B] at induction week 12 in all patients, irrespective of prior biologic experience. For the endoscopic endpoints of response, remission, and ulcer-free endoscopy, achievement of a reduction in SF, reduction in APS, enhanced clinical response, SF remission, and SF/APS clinical remission, as early as week 2, were associated with a significantly greater likelihood of achieving these endpoints at week 12 for patients with prior biologic experience only [Supplementary Figure 3B]. It should be noted that several odds ratios and corresponding 95% CIs were not estimable due to no incidence. For example, all patients without prior bio-failure treated with RZB 600 mg IV achieved clinical remission [per SF/AP] at week 1 and achieved CDAI clinical remission at week 12, and therefore the odds ratios and corresponding 95% CIs were not estimable. Meaningful interpretation of odds ratios with respect to early PROs as predictors of clinical and endoscopic response to maintenance dosing by prior biologic experience was not possible, due to low patient numbers [RZB 180 mg SC: with prior bio-failure, *n* = 49; without prior bio-failure, *n *= 20; RZB 360 mg SC: with prior bio-failure, *n* = 45; without prior bio-failure, *n* = 20].

When examining patients with ileal or colonic disease, the odds ratios for early achievement of PROs and the achievement of week 12 endpoints were generally similar for both subgroups compared with the overall population. Achievement of PROs at at least one of the early time points was associated with an increased likelihood of achieving clinical remission [SF/APS or CDAI] at induction week 12 in both subgroups [Supplementary Figure 4A]. In contrast, early achievement of only a few PROs were significantly associated with achieving the endoscopic endpoints of response, remission, and ulcer-free endoscopy at week 12 in either subgroup [Supplementary Figure 4B]., the only commonality observed between the subgroups was the association of a reduction in SF at week 3 with a greater likelihood of achieving endoscopic remission [Supplementary Figure 4B]. One notable difference includes SF remission at weeks 1, 2, and 3 in patients with colonic, but not ileal, disease being significantly associated with achieving ulcer-free endoscopy at week 12. With respect to the combined endpoint of SF/APS clinical remission and endoscopic response at week 12, both subgroups demonstrated a significantly greater likelihood of achieving this endpoint with early reduced SF; however in patients with colonic disease, early changes in all endpoints [except AP remission] were significantly more likely to achieve the combined endpoint, whereas only week 2 enhanced clinical response, SF remission, and AP remission demonstrated this predictive association at week 12 [Supplementary Figure 4C]. An assessment of early PROs as predictors of response to maintenance dosing by disease location was not possible due to low patient numbers [RZB 180 mg SC: colonic disease, *n *= 28; ileal disease, *n* = 7; RZB 360 mg SC: colonic disease, *n *= 26; ileal disease, *n* = 9].

#### 3.1.4. Safety

A full safety analysis has been published previously.^11,12^ Briefly, RZB induction and maintenance therapy was well tolerated. The incidence of adverse events was low and consistent between treatment arms and between studies. Rates of adverse events leading to treatment discontinuation were low, with higher frequencies observed in the PBO arms compared with RZB arms in the ADVANCE and MOTIVATE studies. The most frequently reported serious AE across the studies was worsening of CD, with lower rates in the RZB arms, or events primarily gastrointestinal in nature, likely reflecting underlying disease. Rates of serious infections and active tuberculosis were low with RZB treatment, with an observed frequency similar to or lower than the PBO arms. No new safety risks with RZB were identified.

## 4. Discussion

Early symptomatic benefits, such as those reported herein, are an important goal of CD management and are an increasingly important consideration when selecting a biologic. Not only are the physical symptoms of CD burdensome for patients, but they interfere with physical function and activities of daily living, and over time they are associated with elevated symptoms of depression and anxiety and decreased hrQoL.^13–18^ Accordingly, current ‘Selecting Therapeutic Targets in Inflammatory Bowel Disease’ [STRIDE] II recommendations now include clinical response and remission as immediate and intermediate targets, and restoration of quality of life as a long-term endpoint, in IBD management.^8^

Anti-tumour necrosis factor [TNF] treatment represents a central treatment modality in CD, but primary non-response and secondary loss of response to this treatment affect ~10–30% and 23–46% of adult CD patients, respectively.^19^ Alternatives to anti-TNFs include biologics with newer mechanisms of action, such as vedolizumab [anti-α4β7 integrin] and ustekinumab [anti-p40], which have been shown to be effective in achieving symptomatic improvement in patients who are naïve or intolerant to or who inadequately respond to anti-TNF treatment.^20–24^ The rapidity of symptom relief among these biologic agents varies however, and comparative studies are lacking. Reports have shown that at week 2 of induction, 38% of patients receiving ustekinumab and 46% of patients receiving adalimumab achieved clinical response (CDAI score decreased by ≥100 points from baseline [CR-100]), and 31.4% of patients receiving vedolizumab achieved clinical response [CR-100] at week 6.^22,25^ Here we show that as early as week 1, a single IV induction dose of RZB [anti-IL23 p19] led to greater improvements in a variety of individual and combined SF and APS PROs compared with PBO, with significant improvements demonstrated by week 2. Symptomatic improvements were observed both in the overall patient population of ADVANCE [mixed population of patients with and without a history of inadequate response or intolerance to one or more biologics, including agents such as vedolizumab and ustekinumab], and in MOTIVATE, a more refractory population including only patients with prior bio-failure.

Of the PROs assessed as predictors of response, most [as early as week 1] were associated with a significantly greater likelihood of achieving clinical, endoscopic, and composite clinical and endoscopic endpoints at induction week 12. Subgroup analyses by previous biologic experience and disease location did not indicate differences in PROs predicting early response to RZB for symptomatic endpoints at week 12. In contrast, for endoscopic endpoints at week 12, PROs were generally only predictive in the prior bio-failure patient subgroup, and early achievement of SF remission in patients with colonic disease was associated with a greater likelihood of achieving ulcer-free endoscopy at week 12. Low patient numbers, especially for patients without prior bio-failure and for patients with ileal disease, hindered meaningful interpretation of the examination of early PROs as predictors of week 52. Nevertheless, early improvement of PROs during IV induction with RZB may have implications for achieving near-term and long-term treatment goals.

Despite the prognostic value of early symptom resolution for achieving post-induction outcomes at week 12, responses to treatment at week 52 are considered most meaningful to modify disease outcome and are recommended as important treatment targets by the STRIDE-II consensus.^8^ In general, we observed that patients who achieved SF remission or AP remission early during induction were more likely to achieve clinical remission [CDAI] with either maintenance dose at week 52, which is aligned with the previous finding that patients with moderate to severe AP and/or SF scores post-induction were less likely to achieve CDAI clinical remission at 1 year.^26^ Our data also demonstrated that only patients who received RZB 360 mg SC in maintenance and who achieved a reduction in SF at week 3 of induction were significantly more likely to achieve the objective endpoints of endoscopic response and ulcer-free endoscopy at week 52, as well as the composite endpoint of endoscopic response + SF/APS clinical remission, but not endoscopic remission. Again, this result corroborates previous reports demonstrating a limited correlation of early resolution of PROs with endoscopic disease activity, as well as no greater likelihood of achieving 1-year endoscopic remission with post-induction PRO improvement vs no improvement.^26–28^ More recent study findings, however, suggest that post-induction resolution of PROs does become prognostic for 1-year combined clinical and endoscopic remission when a patient’s most severely elevated [dominant] PRO is evaluated and that, in this context, SF may be a better prognostic marker of endoscopic disease compared with AP.^29^ Such an approach to PRO evaluation in future analyses may better establish the relationship between early PRO resolution and long-term endoscopic outcomes, and may yield short-term targets that predict meaningful long-term change.

This study has limitations that should be stated. The analyses reported herein are post hoc, and the ADVANCE and MOTIVATE studies were not designed to evaluate the impact of RZB on early symptom improvement within the first 3 weeks of treatment. Due to the long half-life of RZB and its prolonged pharmacodynamic effects, the re-randomised study design of FORTIFY may have affected odds ratios calculations for outcomes at week 52. Also, by nature, PRO measures are subjective and susceptible to reporting bias; however, the qualitative measures of disease activity examined here, which measure symptoms that are among the most burdensome, are relevant to capturing the impact of treatment of greatest importance to patients and could be considered a strength rather than a limitation. It is also important to consider that some patients may have a delayed response to therapy, and identifying predictors of delayed response is of interest and merits further study.^30,31^ Last, meaningful interpretation of findings from examination of PROs as early predictors of response in patient subgroups [with vs without prior bio-failure, or ileal vs colonic disease location] at week 52 was encumbered by low patient numbers.

In summary, this post hoc analysis provides additional support for the utility of RZB therapy in patients with moderately to severely active CD irrespective of prior bio-failure status, with significant benefit attainable after the first induction dose of RZB. No new safety signals with RZB were observed in the ADVANCE or MOTIVATE studies, and the overall safety profile was consistent with the known safety profile of RZB.

## Supplementary Data

Supplementary data are available at *ECCO-JCC* online.

jjad206_suppl_Supplementary_Materials

## Data Sharing Statement

AbbVie is committed to responsible data sharing regarding the clinical trials sponsored. This includes access to anonymised, individual-level, and trial-level data [analysis datasets], as well as other information [eg, protocols, clinical study reports, or analysis plans], as long as the trials are not part of an ongoing or planned regulatory submission. This includes requests for clinical trial data for unlicensed products and indications. These clinical trial data can be requested by any qualified researchers who engage in rigorous, independent, scientific research, and will be provided following review and approval of a research proposal, statistical analysis plan [SAP], and execution of a data sharing agreement [DSA]. Data requests can be submitted at any time after approval in the USA and Europe and after acceptance of this manuscript for publication. The data will be accessible for 12 months, with possible extensions considered. For more information on the process or to submit a request, visit the following link: [https://www.abbvieclinicaltrials.com/hcp/data-sharing/.html].
