# Supplementary material for: Risankizumab Induction Therapy Achieves Early Symptom Improvements That Are Associated With Future Clinical and Endoscopic Outcomes in Crohn’s Disease: Post Hoc Analysis of the ADVANCE, MOTIVATE, and FORTIFY Phase 3 Studies
Source: J Crohns Colitis. 2023 Dec 9;18(6):818–27. doi: 10.1093/ecco-jcc/jjad206 (PMC11147806; doi:10.1093/ecco-jcc/jjad206)
Supplement: jjad206_suppl_Supplementary_Materials [file jjad206_suppl_supplementary_materials.docx]

## Supplementary Materials

## Supplementary Figure 1. ADVANCE Achievement of SF or APS Remission Over Time for Patients With/Without Prior Bio-Failure at Weeks 1, 2, and 3 (ITT Population; NRI-NC)

**A**

**A**

**B**

**B**

**B**

**D**

**B**

**C**

**A**

**B**

PBO, N = 97; RZB 600 mg IV, N = 195 for subjects with prior bio-failure; N = 78; RZB 600 mg IV, N = 141 for subjects without prior bio-failure.

**P* ≤ 0.05, ****P* ≤ 0.001 vs PBO.

AP = Abdominal pain; APS remission = Average daily APS ≤ 1 and not worse than baseline; ITT = intention-to-treat; NRI-NC = non-responder imputation with no special data handling for missing data due to COVID-19; PBO = placebo; RZB = risankizumab; SF = stool frequency; SF remission = Average daily SF ≤ 2.8 and not worse than baseline

## Supplementary Figure 2. ADVANCE Achievement of SF/APS Clinical Remission or SF/APS Enhanced Clinical Response Over Time for Patients with/without Prior Bio-Failure at Weeks 1, 2, and 3 (ITT Population; NRI-NC)

**C**

**A**

**B**

**D**

**B**

**B**

**B**

**A**

**A**

**B**

PBO, N = 97; RZB 600 mg, N = 195 for subjects with prior bio-failure; PBO, N = 78; RZB 600 mg, N = 141 for subjects without prior bio-failure

**P* ≤ 0.05, ***P* ≤ 0.01, ****P* ≤ 0.001 vs. PBO.

APS = Abdominal pain score; Enhanced clinical response per SF/APS criteria = ≥ 60% decrease in average daily SF and/or ≥ 35% decrease in average daily AP (and both not worse than baseline) and/or clinical remission; ITT = Intention-to-treat; NRI-NC = Non-responder imputation with no special data handling for missing data due to COVID-19; PBO = Placebo; RZB = Risankizumab; SF = Stool frequency; SF/APS clinical remission = Average daily SF ≤ 2.8 and average daily AP score ≤ 1 and both not worse than baseline.

## Supplementary Figure 3. Early Clinical Outcomes as Predictors of Achieving Endoscopic and/or Symptomatic Endpoints Following Induction in Patients with or without a History of Prior Bio-Failure

**B**

**A**

RZB 600 mg IV with prior bio-failure, n=386; without prior bio-failure, n = 141; A significant odds ratio less than 1 for ΔSF and ΔAPS is reflective of the fact that as the variable decreases, the event is more likely to occur. AP remission = average daily AP score ≤ 1 and not worse than baseline; SF remission = average daily SF ≤ 2.8 and not worse than baseline; SF/APS clinical remission = average daily SF ≤ 2.8 and not worse than baseline and average daily AP score ≤1 and not worse than baseline; CDAI clinical remission = CDAI < 150; Enhanced clinical response = ≥60% decrease in average daily SF and/or ≥35% decrease in average daily AP score and both not worse than baseline, and/or clinical remission; Endoscopic response = decrease in SES-CD > 50% from baseline (or for subjects with isolated ileal disease and a Baseline SES-CD of 4, at least a 2 point reduction from baseline), as scored by central reviewer; Endoscopic remission = SES-CD ≤ 4 and at least a 2 point reduction versus baseline and no subscore greater than 1 in any individual variable, as scored by a central reviewer; Ulcer-free endoscopy = SES-CD ulcerated surface subscore of 0 in patients with SES-CD ulcerated surface subscore ≥ 1 at baseline, as scored by a central reviewer. **P* ≤ 0.05, ***P* ≤ 0.01, ****P* ≤ 0.001

## Supplementary Figure 4. Early Clinical Outcomes as Predictors of Achieving Endoscopic and/or Symptomatic Endpoints Following Induction in Patients with Ileal or Colonic Disease

**A**

**B**

**A**

**C**

RZB 600 mg IV, with ileal disease, n = 190; with colonic disease, n = 85; A significant odds ratio less than 1 for ΔSF and ΔAPS is reflective of the fact that as the variable decreases, the event is more likely to occur. AP remission = average daily AP score ≤1 and not worse than baseline; SF remission = average daily SF ≤ 2.8 and not worse than baseline; SF/APS clinical remission = average daily SF ≤ 2.8 and not worse than baseline and average daily AP score ≤1 and not worse than baseline; CDAI clinical remission = CDAI < 150; Enhanced clinical response = ≥60% decrease in average daily SF and/or ≥35% decrease in average daily AP score and both not worse than baseline, and/or clinical remission; Endoscopic response = decrease in SES-CD > 50% from baseline (or for subjects with isolated ileal disease and a Baseline SES-CD of 4, at least a 2 point reduction from baseline), as scored by central reviewer; Endoscopic remission = SES-CD ≤ 4 and at least a 2 point reduction versus baseline and no subscore greater than 1 in any individual variable, as scored by a central reviewer; Ulcer-free endoscopy = SES-CD ulcerated surface subscore of 0 in patients with SES-CD ulcerated surface subscore ≥ 1 at baseline, as scored by a central reviewer. **P* ≤ 0.05, ***P* ≤ 0.01, ****P* ≤ 0.001

## Supplementary Table 1. Number (%) of Patients With Missing PRO Scores at Baseline

|  | | **ADVANCE** | | | **MOTIVATE** | | |
| --- | --- | --- | --- | --- | --- | --- | --- |
|  |  | **PBO**  **IV** | **RZB**  **600 mg IV** | **RZB**  **1200 mg IV** | **PBO**  **IV** | **RZB**  **600 mg IV** | **RZB**  **1200 mg IV** |
| **SF n (%)** | **Week 1** | 7 (4.0) | 16 (4.8) | 8 (2.4) | 3 (1.6) | 3 (1.6) | 10 (5.2) |
|  | **Week 2** | 11 (6.3) | 12 (3.6) | 14 (4.1) | 16 (8.6) | 3 (1.6) | 8 (4.2) |
|  | **Week 3** | 16 (9.1) | 17 (5.1) | 11 (3.2) | 11 (5.9) | 5 (2.6) | 9 (4.7) |
| **APS n (%)** | **Week 1** | 7 (4.0) | 16 (4.8) | 8 (2.4) | 3 (1.6) | 3 (1.6) | 10 (5.2) |
|  | **Week 2** | 11 (6.3) | 12 (3.6) | 14 (4.1) | 16 (8.6) | 3 (1.6) | 8 (4.2) |
|  | **Week 3** | 16 (9.1) | 17 (5.1) | 11 (3.2) | 11 (5.9) | 5 (2.6) | 9 (4.7) |

## Supplementary Table 2. Number (%) of Patients With Raised Stool Frequency (SF) or Raised Abdominal Pain Score (APS), or in SF Remission or AP Remission, at Baseline

|  | **ADVANCE** | | | | **MOTIVATE** | | | |
| --- | --- | --- | --- | --- | --- | --- | --- | --- |
|  | **PBO** | **RZB** | **RZB** |  | **PBO** | **RZB** | **RZB** |  |
|  | **IV** | **600 mg IV** | **1200 mg IV** | **Total** | **IV** | **600 mg IV** | **1200 mg IV** | **Total** |
| **Raised SF**  **SF > 2.8, n (%)** | 175  (93.6) | 174  (91.1) | 174  (91.1) | 523 (91.9) | 160  (91.4) | 305  (90.8) | 305  (90) | 770 (90.6) |
| **SF Remission**  **SF ≤ 2.8, n (%)** | 12  (6.4) | 17  (8.9) | 17  (8.9) | 46 (8.1) | 15  (8.6) | 31  (9.2) | 34  (10) | 80 (9.4) |
| **Raised APS**  **APS > 1.0, n (%)** | 170  (90.9) | 174  (91.1) | 173  (90.6) | 517 (90.9) | 159  (90.9) | 303  (90.2) | 301  (88.8) | 763 (89.8) |
| **AP Remission**  **APS ≤ 1.0, n (%)** | 17  (9.1) | 17  (8.9) | 18  (9.4) | 52 (9.1) | 16  (9.1) | 33  (9.8) | 38  (11.2) | 87 (10.2) |
